# The Low‐Resolution Version of HadGEM3 GC3.1: Development and Evaluation for Global Climate

**DOI:** 10.1029/2018MS001370

**Published:** 2018-11-14

**Authors:** Till Kuhlbrodt, Colin G. Jones, Alistair Sellar, Dave Storkey, Ed Blockley, Marc Stringer, Richard Hill, Tim Graham, Jeff Ridley, Adam Blaker, Daley Calvert, Dan Copsey, Richard Ellis, Helene Hewitt, Patrick Hyder, Sarah Ineson, Jane Mulcahy, Antony Siahaan, Jeremy Walton

**Affiliations:** ^1^ National Centre for Atmospheric Science, Department of Meteorology University of Reading Reading UK; ^2^ National Centre for Atmospheric Science, School of Earth and Environment University of Leeds Leeds UK; ^3^ Met Office Hadley Centre Exeter UK; ^4^ National Oceanography Centre Southampton UK; ^5^ Centre for Ecology and Hydrology Wallingford UK; ^6^ Now at the British Antarctic Survey Cambridge UK

**Keywords:** coupled climate model, Earth system model, HadGEM, model evaluation, CMIP6

## Abstract

A new climate model, HadGEM3 N96ORCA1, is presented that is part of the GC3.1 configuration of HadGEM3. N96ORCA1 has a horizontal resolution of ~135 km in the atmosphere and 1° in the ocean and requires an order of magnitude less computing power than its medium‐resolution counterpart, N216ORCA025, while retaining a high degree of performance traceability. Scientific performance is compared to both observations and the N216ORCA025 model. N96ORCA1 reproduces observed climate mean and variability almost as well as N216ORCA025. Patterns of biases are similar across the two models. In the northwest Atlantic, N96ORCA1 shows a cold surface bias of up to 6 K, typical of ocean models of this resolution. The strength of the Atlantic meridional overturning circulation (16 to 17 Sv) matches observations. In the Southern Ocean, a warm surface bias (up to 2 K) is smaller than in N216ORCA025 and linked to improved ocean circulation. Model El Niño/Southern Oscillation and Atlantic Multidecadal Variability are close to observations. Both the cold bias in the Northern Hemisphere (N96ORCA1) and the warm bias in the Southern Hemisphere (N216ORCA025) develop in the first few decades of the simulations. As in many comparable climate models, simulated interhemispheric gradients of top‐of‐atmosphere radiation are larger than observations suggest, with contributions from both hemispheres. HadGEM3 GC3.1 N96ORCA1 constitutes the physical core of the UK Earth System Model (UKESM1) and will be used extensively in the Coupled Model Intercomparison Project 6 (CMIP6), both as part of the UK Earth System Model and as a stand‐alone coupled climate model.

## Key Points

- A low‐resolution, traceable version of the current Met Office Hadley Centre climate model HadGEM3 GC3.1 is presented
- The scientific performance is comparable to the medium‐resolution version, while requiring much less computational resources
- In the low‐resolution version the Southern Ocean warm bias is reduced, linked with a more realistic ocean circulation

## 1 Introduction

Comprehensive and detailed models of the global climate system, called atmosphere‐ocean general circulation models (AOGCMs), are essential tools to understand the climate system. Such an understanding strives to explain past, present, and future climate processes and their changes. This is especially relevant in the context of ongoing man‐made global change (IPCC, 2013). For the current global intercomparison of AOGCMs in the Coupled Model Intercomparison Project Phase 6 (CMIP6; Eyring et al., 2016), it is expected that simulations from several dozen AOGCMs will be submitted for analysis. The same models will also be used to make projections of the future climate over the coming century, providing a vital service to policy‐makers and society at large.

The physical climate model HadGEM3 (Hadley Centre Global Environment Model 3) has been developed by the Met Office Hadley Centre in the United Kingdom since the late 2000s. It was always intended that it could be configured at different spatial resolutions and with varying degrees of complexity (e.g., number of physical and biogeophysical processes included) depending on the research questions (Hewitt et al., 2011). Here we report on a new configuration, developed at a relatively coarse horizontal resolution, N96ORCA1, for use as the physical core of the UK Earth System Model (UKESM1). Because of the added process complexity in an Earth system model (ESM), and the need to advect many additional tracers, the previously existing medium‐resolution version of HadGEM3, N216ORCA025 (Williams et al., 2017) would have been computationally too expensive for this purpose. The horizontal resolutions are 135 km in the atmosphere and 1° in the ocean for N96ORCA1 and 60 km/0.25° for N216ORCA025.

In this article we give a first description of the physical low‐resolution configuration N96ORCA1, comprising atmosphere, ocean, sea ice, and land surface models. Within this special issue of *JAMES* on HadGEM3 GC3 and UKESM1, there are, or will be, a number of companion papers exploring in greater depth many of the aspects that we mention here. For instance, Williams et al. (2017) describe the medium‐resolution atmosphere, ocean, sea ice, and land surface model configuration. A forthcoming paper will describe the performance of UKESM1, which is based on N96ORCA1 and represents interactively the global carbon cycle, including dynamic vegetation and a model for stratosphere‐troposphere chemistry. Further papers in preparation will evaluate the performance of the preindustrial (PI) control simulations, the historical simulations for CMIP6, radiative forcing and climate sensitivity, etc.

In the process of developing and evaluating the coarse‐resolution version, N96ORCA1, in relation to the preexisting resolutions, N216ORCA025 and N96ORCA025, *traceability* was of paramount importance. By traceability we mean that ideally the scientific settings and adjustments of HadGEM3 are not dependent on the chosen spatial resolution. Excepted are parameterizations of processes that are resolved at higher resolution, for example, mesoscale ocean eddies that need to be fully parameterized at 1° resolution but less so at 0.25° resolution. For any resolution‐specific settings that are necessary, we have documented their motivation and their consequences carefully in the present paper. At the same time, we aimed at reproducing the observed, present‐day (PD) climate as closely as possible.

Changing the spatial resolution of a model can affect the scientific performance. For instance, Lauer et al. (2018) showed an improvement in—mostly intraday—rainfall variability over tropical regions with increasing resolution of the EC‐Earth atmosphere model (up to, but not beyond, ~0.4°). For diagnostics related to mean precipitation, Demory et al. (2014) found an improvement over the same range of resolutions in an earlier version of HadGEM3. At the same time, as we show in this paper (for the Southern Ocean), an increase in resolution does not necessarily lead to improved model performance.

Model biases (affecting mean values or variability) can have an effect on the future climate response, as assessed by evaluation of historical simulations against observations (e.g., Massonnet et al., 2012). Sometimes a relationship between model biases and future feedback responses, common across an ensemble of models, can be exploited to constrain the future response of a given process (e.g., Wenzel et al., 2014). However, it is not a given that a reliable model performance during the observational record equates to a realistic future projection response (Knutti et al., 2010). In addition, there are a number of long‐standing biases, common to many climate models, that may predicate an unrealistic future response compared to what would be seen if the bias were corrected through physically reasonable improvements. These include sea ice biases and impacts on the future sea ice albedo feedback (Qu & Hall, 2007), biases in surface‐atmosphere interaction and the magnitude of future soil moisture feedbacks (Hohenegger et al., 2009), and systematic PD cloud biases influencing future cloud feedbacks (Trenberth & Fasullo, 2010). By analyzing such biases (and the impact of different spatial resolutions) we hope to lead the way to their further understanding and reduction.

The component models of HadGEM3 are the Unified Model (UM) for the atmosphere, Nucleus for European Modeling of the Ocean (NEMO) for the ocean, CICE for the sea ice, and JULES for land surface processes. Hewitt et al. (2011) provide a detailed account of how these component models interact through the OASIS3‐MCT coupler. The present low‐resolution configuration of HadGEM3 is based on the latest release of the global coupled configuration, GC3.1 (Williams et al., 2017). This comprises the latest configurations for the atmosphere, GA7.1 (Walters et al., 2017); the ocean, GO6.0 (Storkey et al., 2018); the sea ice, GSI8.1 (Rae et al., 2015; Ridley et al., 2018); and the land surface, GL7.0 (Walters et al., 2017). Both the atmosphere and the ocean models use a common vertical resolution across different horizontal resolutions (GA7.1: 85 levels, GO6.0: 75 levels).

The following section describes the development process of N96ORCA1 and details how traceability across resolutions was ensured. The bulk of this paper is dedicated to evaluating the model in section 3, beginning with the global radiation budget and progressing from the ocean, the sea ice, the atmosphere, and the land surface to coupled atmosphere‐ocean variability. We present our conclusions in section 4.

## 2 Development and Traceability

The development of HadGEM3 GC3.1 N96ORCA1 started by changing the ocean resolution of the N96ORCA025 configuration as maintained by the Met Office Hadley Centre. The NEMO ocean model (Madec and the NEMO Team, 2016) has a shared configuration at 1° resolution dubbed *shaconemo* (available from http://forge.ipsl.jussieu.fr/shaconemo). This configuration, with minor variations, is employed by four other ESMs across Europe. In order to maintain comparability with these ESMs, we employed the grid size‐dependent settings of this ORCA1 configuration, as detailed in Table 1, while fully making use of new and improved parameterizations (generally not grid size dependent) of the UK GO6 configuration of NEMO (Storkey et al., 2018). These include a prescribed field of turbulent kinetic energy (TKE) at the ocean bottom at a few choke points (e.g., the Indonesian throughflow) and an asymmetrical latitudinal profile for the ad hoc parameterization of the penetration of TKE below the mixed layer (based on Gaspar et al., 1990). We use climatological meltwater input from ice shelves throughout their depth and iceberg calving flux as described in Williams et al. (2017).

**Table 1:** Traceability Between the N96ORCA1 and N216ORCA025 Configurations of HadGEM3 GC3.1

Model component: setting	Value in N96ORCA1	Value in N216ORCA025	Comment
OASIS: coupling frequency	3 hr	1 hr	

NEMO: horizontal viscosity	Laplacian, 20,000 m^2^/s	Bi‐Laplacian, −1.5 × 10^−11^ m^4^/s	Nominal value

NEMO: coefficient for horizontal viscosity	3‐D field	2‐D field	3‐D field in ORCA1 to help resolving equatorial undercurrents

NEMO: parameterization of mesoscale eddies	Used (Held & Larichev, 1996)	Not used	

NEMO: isopycnal diffusion coefficient	1,000 m^2^/s	150 m^2^/s	

NEMO: geothermal heat flux climatology	Goutorbe et al. (2011)	Stein and Stein (1992)	Impact is discernible but small

NEMO grid: type of north fold	*F* point (vorticity)	*T* point (tracer)	

NEMO: time step	45 min	20 min	

CICE: albedo for snow on sea ice	albsnowv_cice = 0.96albsnowi_cice = 0.68	albsnowv_cice = 0.98albsnowi_cice = 0.70	Compensation for deficient transport of warm Atlantic water into the Arctic in ORCA1.

UM: time step	20 min	15 min	

UM: us_am	1.45	1.4	Multiplicative u* correction for dust aerosols affects the relationship between the amount of dust lifted from the land surface and the wind speed.

UM: ussp_launch_factor	1.3	1.2	Gravity wave drag: factor enhancement for wave launch amplitude

*Note*. NEMO = Nucleus for European Modeling of the Ocean; UM = Unified Model.

An overview of the differences between N96ORCA1 and N216ORCA025 is given in Table 1. The bulk of the differences are considered best practice when changing the horizontal resolution in a coupled general circulation model (GCM). With the coarser resolution, a longer time step can be employed in both ocean and atmosphere. Concomitantly, the coupling frequency is reduced while still resolving the diurnal cycle. ORCA1's resolution is not eddy permitting. Hence, a parameterization for transport by mesoscale eddies is necessary (based on Gent et al., 1995, with a spatially varying coefficient following Held and Larichev, 1996), and the parameterization for isopycnal mixing (Redi, 1982) needs to use a larger coefficient than ORCA025. In addition, bi‐Laplacian viscosity to suppress small‐scale patterns is not needed, and Laplacian viscosity is used instead. The vertical resolution is the same for the two configurations, with 75 levels whose thickness increases with depth, from 1 m at the surface to about 200 m at the bottom.

The tripolar ORCA1 grid has its north fold (the line that connects the two grid poles in the Northern Hemisphere [NH]) on vorticity points, in contrast to ORCA025 where it is along tracer points. The ORCA025 configuration as part of GO6 uses an older climatology for the geothermal heat fluxes (Stein & Stein, 1992) with values about twice as large as in the more recent one from *shaconemo* (Goutorbe et al., 2011). The only impact of note is a 5% decrease in the rate of globally volume‐averaged warming (not shown) in a PD control simulation when using the Goutorbe et al. (2011) climatology instead of the older one.

In the GO6 configurations it was found that in small inlets at the coast of Antarctica that have a size of only one grid point excessive sea ice can build up over time. This is a combined effect of meltwater input and lack of advection in single‐grid point areas, specifically on the grid employed by CICE (a different type of grid than NEMO). While the stability of the model is unaffected by this, we decided to smooth the coastline of Antarctica, closing these inlets, to avoid this effect altogether.

The only intentional change made to the physical setup of ORCA1, relative to ORCA025, is the use of a slightly lower albedo for snow on sea ice. As explained in detail in section 3.3, we introduced this change to compensate for too weak sea ice bottom melt in the Arctic, itself arising from deficient advection of warm Atlantic water through Fram Strait. In the development process we varied a number of NEMO parameters with the aim to decrease the sea surface temperature (SST) cold bias in the northwest Atlantic discussed below. These tests had little impact on the cold SST bias; a brief overview of them and their outcome is given in Appendix A.

Coming back to Table 1, there are a small number of resolution‐dependent changes in the UM between the higher‐resolution N216 and the N96 used here. Apart from the longer time step mentioned above, the lower maximum wind speed in N96 means that the parameter that affects how much dust is picked up by the wind is increased slightly in N96 to compensate. In a similar vein, since at N96 fewer gravity waves are resolved, the parameter affecting the wave launch amplitude is increased slightly in N96 too.

In terms of computational performance, N96ORCA1 currently produces an output of about 2.3 model years per wall clock day (ypd) on the Met Office Cray using 13 nodes, or 6.5 ypd using 75 nodes (1 node has 36 cores). The UM alone requires about 85% of the nodes. Efforts to increase this performance are under way.

## 3 Evaluation and Scientific Performance

The scientific evaluation of HadGEM3 GC3.1 N96ORCA1 we present here covers a long simulation (752 years) under PD forcing (2000 AD). It starts from the EN4 climatology, an objective analysis product based on observational data (Good et al., 2013), using a 1995–2014 average. For the atmosphere and the sea ice, initial conditions from PD model simulations at the previous version of HadGEM3 (GC3.0) are used. For reference and comparison we use a PD simulation (297 years long) from N216ORCA025 with nearly identical initial conditions (Williams et al., 2017). In doing so, we ensure that the present paper documents the traceability between N96ORCA1 and N16ORCA025.

In the following, the analysis focuses on the comparison of the PD simulations with observational data. The alternative to using PD simulations for evaluating a coupled climate model against observations is to run a full set of so‐called historical simulations that include time‐varying forcing over the 165 years up to 2015. This exercise requires a fully spun‐up PI control simulation to be completed in advance of running an ensemble of historical simulations. At the point of documenting our coupled model, such a spin‐up and PI control run had not yet been completed. Furthermore, if a climate model is developed using historical simulations within the development cycle, there is a risk of overtuning toward the observed historical record, which includes both natural climate variability and human forced climate change. Given that models such as HadGEM3‐GC3.1 are developed primarily for investigating the future anthropogenically forced climate change, we chose to avoid this risk and allow the historical climate evolution to be an emergent property of the model rather than a tuned component.

Because the N96ORCA1 configuration forms the core of UKESM1, we will, for each model component, analyze the typical features of a physical GCM as well as circulation features particularly relevant from an ESM perspective. Part of the analysis deals with the transient behavior to understand how the two models drift from the initial conditions toward their own individual equilibrium states. The bulk of the analysis presents the average of years 50–100, after the first 50 years of spin‐up, to assess how the models represent the PD climate.

### 3.1 Top of Atmosphere Radiation Budgets

#### 3.1.1 Global Mean

We begin by considering the time‐dependent global and hemispheric radiation balance on centennial time scales, including surface and planetary albedo. Since our simulations have a continuous year 2000 forcing, these time series are not meant to reproduce observed variability. Rather, we believe that this analysis is instructive to understand the evolving and differing biases in N96ORCA1 and N216ORCA025.

**Figure 1:**
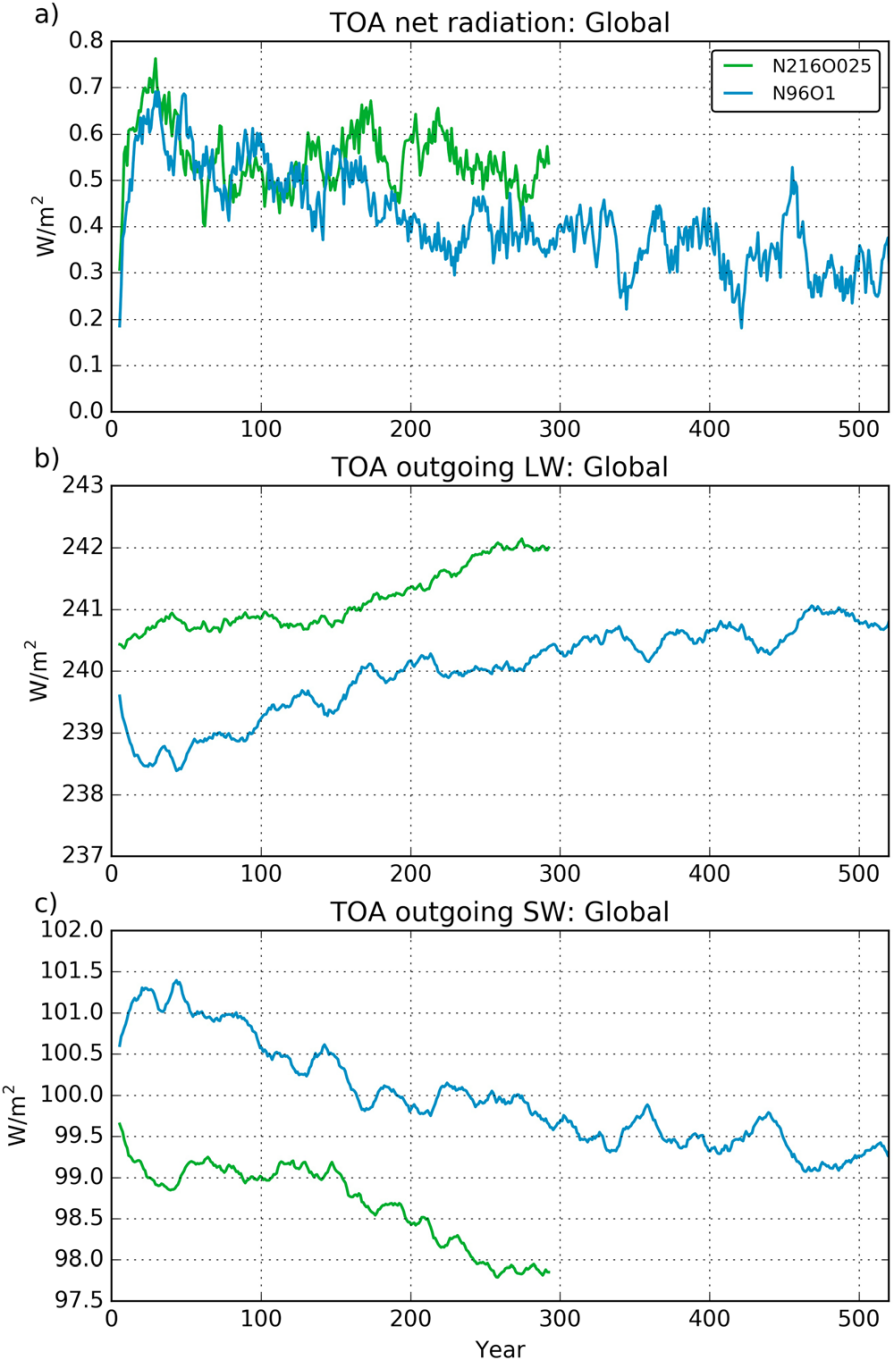
Global radiation budgets at the top of the atmosphere (TOA) for (a) net incoming radiation (downward positive), (b) outgoing longwave (upward positive), and (c) outgoing shortwave radiation (upward positive), from the N96ORCA1 (blue) and N216ORCA025 (green) simulations. The graphs are 11‐year running means.

Figure 1a shows how the N96ORCA1 model, starting from PD conditions, reaches a temporally stable top of atmosphere (TOA, at 85 km) net radiation balance between +0.3 and +0.4 W/m^2^ after 300 to 400 years. The N216ORCA025 model appears to reach an equilibrium somewhat earlier (after about 150 to 200 years) and at a more positive value (+0.5–0.6 W/m^2^) that is closer to observed estimates for recent decades (Allan et al., 2014). As well as analyzing total net radiation, it is also important to consider the shortwave and longwave components of the TOA radiation budget as these may tell a different story to the net radiation.

Figures 1b and 1c show the evolution of TOA outgoing longwave radiation (OLR) and outgoing shortwave radiation (OSR) in the two models. While both start from almost identical initial states, they quickly diverge in terms of global mean OLR. In N96ORCA1 OLR initially drops during the first 20–40 years, as the ocean mixed layer cools relative to its initial conditions. After this adjustment, OLR slowly increases in N96ORCA1 in line with a gradual increase in global mean SST (see Figure 10), stabilizing after ~400 years. In contrast, the N216ORCA025 model does not show an initial drop in OLR (and a much smaller drop in global mean SST; see Figure 10); rather, there is an immediate increase in OLR. This different behavior over the first ~20–40 simulation years is due to the rapid development of a warm upper ocean/SST bias in the N216ORCA025 model across the Southern Ocean, with spatial mean SSTs increasing by ~1.3 °C in the first 30 years of simulation (not shown). This results in a dipolar pattern in SST biases (relative to observations), warm over the Southern Ocean balancing cold elsewhere with respect to global mean values and, therefore, no initial adjustment in either global mean SST or OLR. There is a tendency for the Southern Ocean surface to also warm in N96ORCA1 but at a significantly slower rate than N216ORCA025. By year 30, N96ORCA1 Southern Ocean mean SSTs are 0.3 °C warmer than the initial conditions and 1 °C cooler than N216ORCA025. In terms of global mean SST, the weaker Southern Ocean warming in N96ORCA1 does not balance the cooling SST trend over the rest of the globe and an adjustment of global mean SST and OLR does occur. Beyond the first ~40 years, both models exhibit a gradual increase in global mean OLR. There is a suggestion that the N216ORCA025 OLR stabilizes around year 275, at a value of ~242 W/m^2^, although the period of stable OLR is too short to be conclusive. What is clear is that the apparent stabilization of net TOA radiation from year ~150 onward in N216ORCA025 is not a result of the TOA radiation components stabilizing but rather due to near cancellation of opposite signed trends in global mean OLR and OSR.

As with OLR, global mean OSR also shows opposite initial trends between N96ORCA1 (increasing over the first 20–40 years) and N216ORCA025 (decreasing over the same period). The increase in N96ORCA1 is associated with an increase in sea ice cover during this adjustment period, as the ocean loses heat (cools). This is seen in the increase in surface albedo in that model (Figure 3c), which primarily occurs in the NH (Arctic sea ice increasing, Figure 3d). The inverse (negative) OSR trend in N216ORCA025 is connected to a rapid decrease in surface albedo (Figure 3), with this primarily occurring in the Southern Hemisphere (SH; Antarctic sea ice loss) as a consequence of the warm SST bias in N216ORCA025 over the Southern Ocean. After this adjustment period, both models exhibit a gradual decrease in OSR, again associated with a decrease in surface albedo, with the trend being stronger in N216ORCA025, primarily in the SH.

A continuous decrease in sea ice (and therefore planetary albedo) is expected in a coupled simulation using continuous year 2000 forcing, as this model will have a positive net TOA radiation balance (as seen in Figure 1). It is for this reason we use the period 50–100 years into the respective simulations to compare against observations. This period is after the initial ocean mixed layer adjustment (in N96ORCA1) but early enough in the simulation so the integrated forcing of the model will be similar to that experienced by the real climate system during the period ~1980 to 2010, when the bulk of observations have been made. Over the first 50–100 years of the two simulations OLR and OSR are within the uncertainty range of observations in Loeb et al. (2009), Stephens et al. (2012), and L'Ecuyer et al. (2015) suggesting the model experiences a radiative forcing representative of recent decades.

#### 3.1.2 Hemispheric Means and Hemispheric Gradients

**Figure 2:**
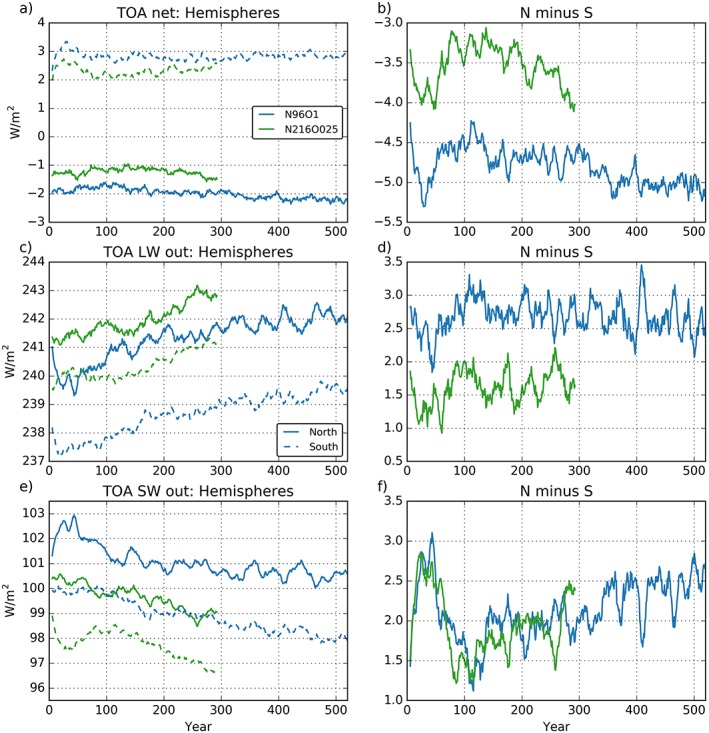
(a, c, e) Global radiation budgets at the top of atmosphere (TOA) for either hemisphere (Northern Hemisphere: solid; Southern Hemisphere: dashed). (b, d, f) Same for the hemispheric difference (Northern Hemisphere minus Southern Hemisphere). Otherwise as Figure 1. Note that for net radiation (panels a and b) downward radiation is positive, while for outgoing radiation (the other panels) upward radiation is positive. LW = longwave; SW = shortwave.

Earth loses energy in the NH (Figure 2a, solid lines) and gains energy in the SH (dashed lines). As a consequence, an interhemispheric, northward energy transport is required (Marshall et al., 2013). In terms of TOA net radiation this NH to SH gradient is ~1.2 W/m^2^ more negative in N96ORCA1 (about −4.6 W/m^2^) than in N216ORCA025 (about −3.4W/m^2^; see Figure 2b). This results from (i) the SH receiving more radiation in N96ORCA1 (dashed lines in Figure 2a) and (ii) the NH losing more energy at TOA (full lines in Figure 2a) in N96ORCA1. To evaluate the hemispheric gradients of TOA radiation and better understand the cause of differences between the two models, we look at the hemispheric components and gradients of both OSR and OLR.

Stephens et al., 2016 and Voigt et al. (2013) show that observed SH and NH mean OSR are almost equal (e.g., central estimates in Stephens et al., 2016, are SH OSR 99.7 W/m^2^ and NH OSR 99.6 W/m^2^), while the NH to SH OLR gradient is ~1.3 W/m^2^ (Stephens et al., 2016; SH OLR 238.9 W/m^2^, NH OLR 240.2 W/m^2^; although is important to note that observed estimates of OSR and OLR are subject to an uncertainty of ±2 to 3 W/m^2^). Considering OSR, in both hemispheres N216ORCA025 is 1–2 W/m^2^ less reflective than N96ORCA1, with the SH OSR likely more accurate in N96ORCA1 and the NH OSR more accurate in N216ORCA025 (cf. sea ice extent in Figure 15). Put another way, the N216ORCA025 model is not sufficiently reflective to solar radiation in the SH, where N96ORCA1 performs better. In the NH the N96ORCA1 model is likely too reflective and N216ORCA025 is more accurate. Nevertheless, both models have very similar NH to SH OSR gradients of ~1.5 to 2 W/m^2^ (NH more reflective than SH), in contrast to the near‐zero gradient in observations. Problems simulating the hemispheric gradient in OSR have been documented in numerous studies (e.g., Trenberth and Fasullo, 2010; Bodas‐Salcedo et al., 2012; Hwang and Frierson, 2013) and can result both from errors in simulating climatological cloud‐radiation interactions over the Southern Ocean, particularly model underestimates of the prevalence of supercooled liquid clouds in the region (Bodas‐Salcedo et al., 2016; Kay et al., 2016), and from biases in simulated historical anthropogenic aerosol radiative forcing, which is primarily a NH phenomenon (Ghan et al., 2016; Myhre et al., 2013; Regayre et al., 2018). How much, one or both, of these problems contributes to the OSR error in HadGEM3‐GC3.1 requires further analysis.

With respect to hemispheric OLR, the evolution of NH mean OLR is rather similar in both models, with slightly higher values in N216ORCA025. The models differ more in the SH, where after 100 years, N216ORCA025 OLR is ~2 W/m^2^ greater than N96ORCA1 (240 vs. 238 W/m^2^, with a central observational estimate in Stephens et al., 2016, of 238.9 W/m^2^). The N216ORCA025 model (as shown in Figure 9) develops a warm Southern Ocean SST bias, which is partly due to an underestimate of SH atmospheric reflectivity and an associated overestimate in surface downwelling solar radiation (i.e., simulated OSR in N216ORCA025 is ~98 W/m^2^ compared to a central observed estimate of ~99.7 W/m^2^). A positive bias in SH OLR is consistent with (and also a consequence of) such a Southern Ocean warm bias. Over the NH N216ORCA025 OLR is at the upper end of the observed range (~242 W/m^2^ at year 100 compared to a central observed estimate of 240.2 W/m^2^). The hemispheric OLR gradient in N216ORCA025 is therefore quite accurate but results from both hemispheres emitting too much OLR. In contrast, N96ORCA1 is closer to the central observed OLR estimates for both the NH and SH, slightly overestimating in the NH and underestimating in the SH, leading to a hemispheric gradient that is ~1 W/m^2^ more positive than N216ORCA025 and observations.

The combination of similar hemispheric OSR gradients, but different OLR gradients, leads to the difference in hemispheric gradient (NH minus SH) of net TOA radiation between the two models: −4.6 (N96ORCA1) and −3.4 W/m^2^ (N216ORCA025), compared to a central observed estimate of −1.8 W/m^2^. The bulk of these errors against observations are associated with a common positive bias in the NH minus SH OSR gradient, which may have different drivers in the two models (i.e., SH atmosphere not sufficiently reflective in N216ORCA025, NH atmosphere too reflective in N96ORCA1). The more realistic (weaker) net TOA radiation gradient in N216ORCA025 arises from a weaker (more realistic) NH minus SH OLR gradient, which unfortunately results from the N216ORCA025 SH emitting OLR too strongly due to the warm surface Southern Ocean bias.

**Figure 3:**
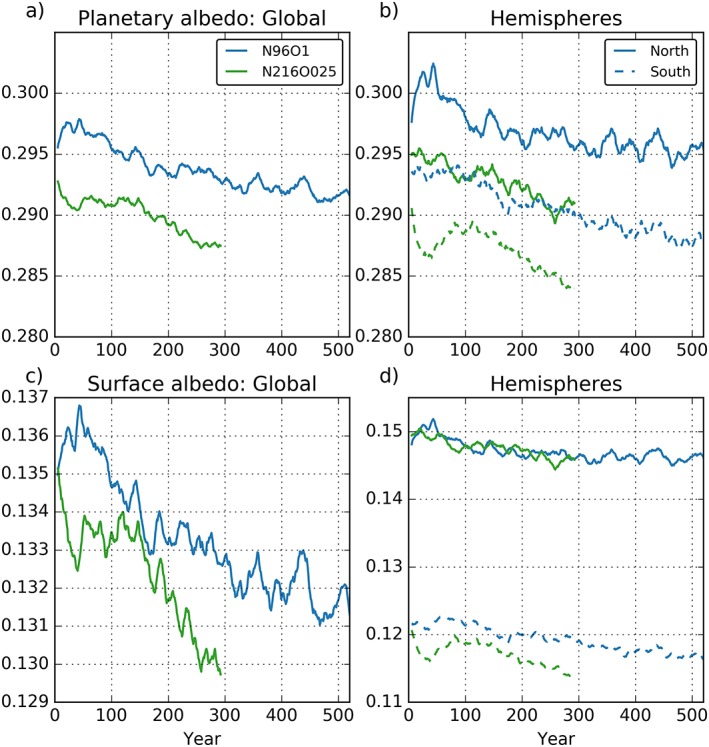
Time series (11‐year running means) of (a) the global planetary albedo at the top of the atmosphere (TOA) and (c) the global surface albedo. Same quantities for either hemisphere shown in (b) and (d). Color coding as in Figure 2.

Total planetary albedo (*α*_p_) in the period 50–100 years of the two simulations (Figure 3a) bounds the observational estimate (*α*_p_ = 0.293; Stephens et al., 2015). Both models show a decrease in planetary albedo, with the N216ORCA025 model consistently lower by ~0.5%. A significant fraction of this trend in planetary albedo can be attributed to decreases in surface albedo, associated with loss of sea ice and snow cover due to the positive net TOA radiation balance in both simulations. This decrease is greater in N216ORCA025, mainly due to a greater decrease in the SH associated with the surface ocean warm bias in that model. Considering hemispheric albedos, the NH surface albedos (Figure 3d) are quite similar in both models, while the NH planetary albedo is clearly higher in N96ORCA1. This is consistent with the N96ORCA1 NH OSR being 1–2 W/m^2^ greater than in N216ORCA025 (Figure 2e) and suggests the OSR difference is primarily due to atmospheric (i.e., cloud‐aerosol‐solar radiation) differences between the two models.

### 3.2 Ocean

For the direct comparison with observations in this and the following subsections we use averages over the simulation years 50 to 100, as discussed in section 3.1.2, omitting the initial short‐term, decadal‐scale adjustment.

**Figure 4:**
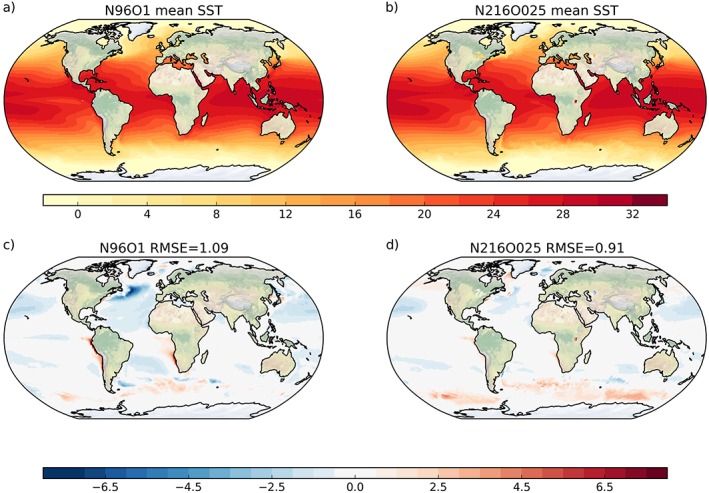
Annual mean simulated SST in (a) N216ORCA025 and (b) N96ORCA1 and the bias against ESA CCI satellite observations for (c) N216ORCA025 and (d) N96ORCA1. Temperature scale in degrees Celsius. Average over year 50 to 100 of the simulations. RMSE = root‐mean‐square error of the simulated field against the observations; SST = sea surface temperature.

A cold bias in the SST develops in the first 20 years of the PD simulation of N96ORCA1. In the global average the SST in N96ORCA1 is about 0.4 K lower than observed (Climate Change Initiative SST; Merchant et al., 2014). There is a distinct geographical pattern to this SST bias (Figure 4c). The cold bias is strongest in the midlatitudes of the NH, while in the Southern Ocean there is actually a warm bias of up to 2 K in the region south of the Antarctic Circumpolar Current (ACC). These patterns are found in many of the CMIP5 climate models (see Figure 9.2b in Flato et al., 2013).

In N216ORCA025 the globally averaged SST cold bias is much smaller, just above 0.1 K. This is linked with the larger Southern Ocean warm bias (Figure 4d), while the NH cold bias is smaller in N216ORCA025 and reduces the root‐mean‐square error in comparison to observations. Williams et al. (2017) provide a more detailed discussion of those large‐scale SST biases in N216ORCA025.

There are a few distinct regional cold‐spot features in the SST in N96ORCA1. Notably the northwest Atlantic is too cold by up to 6 K. This is a known issue in ocean GCMs of 1° grid resolution (Danabasoglu et al., 2014), probably because this resolution is too coarse to correctly represent the poleward and eastward direction of the North Atlantic Current; the current appears too zonal (cf. the central North Atlantic SST contours in Figures 4a and 4b). On a smaller spatial scale, similar problems with representing the position of the Kuroshio and the Brazil Current are discernible in Figure 4c; these appear in a forced, ocean‐only simulation as well (Storkey et al., 2018; Figure 16), indicating that the reason lies in the ocean model rather than with the surface buoyancy fluxes.

Note that the cold bias in most of the low‐latitude to midlatitude Northern Pacific is very similar across the two resolutions in the coupled simulations (Figure 4) but is not found in forced simulations (Storkey et al., 2018), pointing to resolution‐independent causes related to the coupling to the UM. The warm surface bias in subtropical regions of coastal upwelling (west coasts of South America and Southern Africa) is larger in N96ORCA1. It is likely that the representation of this upwelling is improved at higher resolution.

It is worth noting that, while N96ORCA1 has a lower SST than N216ORCA025 at all latitudes on average, the zonal mean net heat flux into the ocean, diagnosed from atmosphere‐only simulations, is roughly the same for N96 and N216 (with minor differences in the tropics and the high northern latitudes). Preliminary analyses suggest that the varying extent of the Southern Ocean warm bias may be related to a resolution dependence in the spin‐up of the coupled ocean‐sea ice‐wind system (and associated ocean heat transports in the initial 5 years of the run) into different stable states. This resolution dependence is not evident in consistent, forced ocean‐only runs, strongly suggesting that it is a coupled phenomenon. Further investigation of the Southern Ocean warm bias in our models is under way.

**Figure 5:**
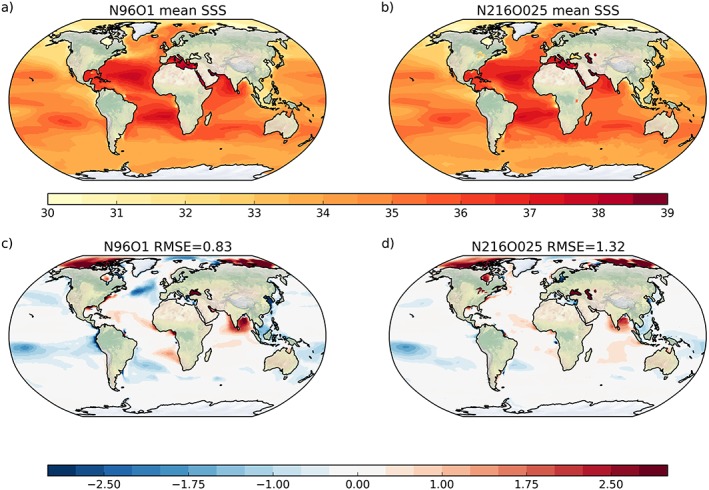
Annual mean simulated SSS in (a) N216ORCA025 and (b) N96ORCA1 and the bias against the EN4 climatology for (c) N216ORCA025 and (d) N96ORCA1. Salinity is measured on the practical salinity scale. SSS = sea surface salinity; RMSE = root‐mean‐square error.

For sea surface salinity (SSS) there are some marked regional biases in N96ORCA1 (Figure 5c), while on the global average the bias against the EN4 climatology (1995–2014 average; Good et al., 2013) is only −0.16. The pattern in N216ORCA025 (Figure 5d) is very similar, notably the strong salty bias in the Bay of Bengal and the fresh biases in the subtropical Pacific on both hemispheres. To some degree, these regional biases also appear in ocean‐only, forced simulations (Storkey et al., 2018), indicating that they occur independent of coupled feedbacks.

The strong fresh bias in the midlatitude North Atlantic, by contrast, occurs only in N96ORCA1. The full simulated SSS (Figure 5a) indicates a freshwater tongue extending directly eastward from the Grand Banks. This is not visible in N216ORCA025 (Figure 5b). It is tempting to conclude that, for this reason, this SSS bias has—directly or indirectly—the same cause as the SST bias in the northwest Atlantic discussed above. Note, however, that the SSS bias minimum is at a more eastern location than the SST bias minimum.

**Figure 6:**
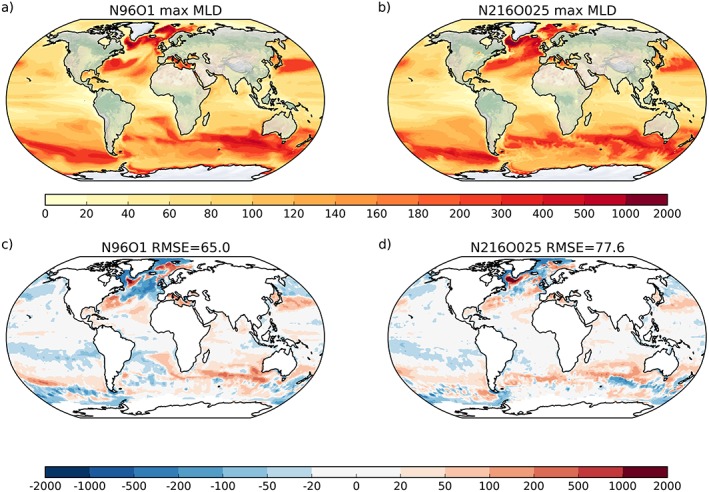
Average maximum monthly mixed layer depth (MLD) in (a) N216ORCA025 and (b) N96ORCA1, in meters, and the bias in (c) N216ORCA025 (left) and (d) N96ORCA1 (right) in the present‐day simulations, shown as the difference against the observational climatology of mixed layer depth (de Boyer Montegut et al., 2004). In the observations, the maximum MLD is not defined in regions of frequent sea ice cover.

The maximum monthly mixed layer depth (MLD) is calculated from monthly mean model output. MLD indicates where water masses are ventilated and deep water is formed, both of which are also relevant for the nutrient distributions in the Earth system context. The maximum MLD occurs in different months across the globe, for instance, in the high latitudes usually at the end of winter on either hemisphere. Figures 6a and 6b show the maximum MLD from both models, while the bias against observations is shown in Figures 6c and 6d. There are notable differences between the two models. For N96ORCA1, in the northwestern Atlantic the region of the cold and fresh bias stands out with a too small maximum MLD. The maximum MLD tends to be too large in the subtropical latitudes on both hemispheres (including poleward of the ACC) and in the deepwater formation regions of the North Atlantic; in large parts of the Pacific (northern midlatitude, southern tropical) they are too small. In the northern North Atlantic, there is a significant difference in that N96ORCA1 has a very deep mixed layer in the central Nordic Seas, as opposed to N216ORCA025 which, by contrast, shows a deeper mixed layer in larger parts of the Labrador and Irminger Seas. This might point to different preferred deepwater formation regions in the two model versions.

**Figure 7:**
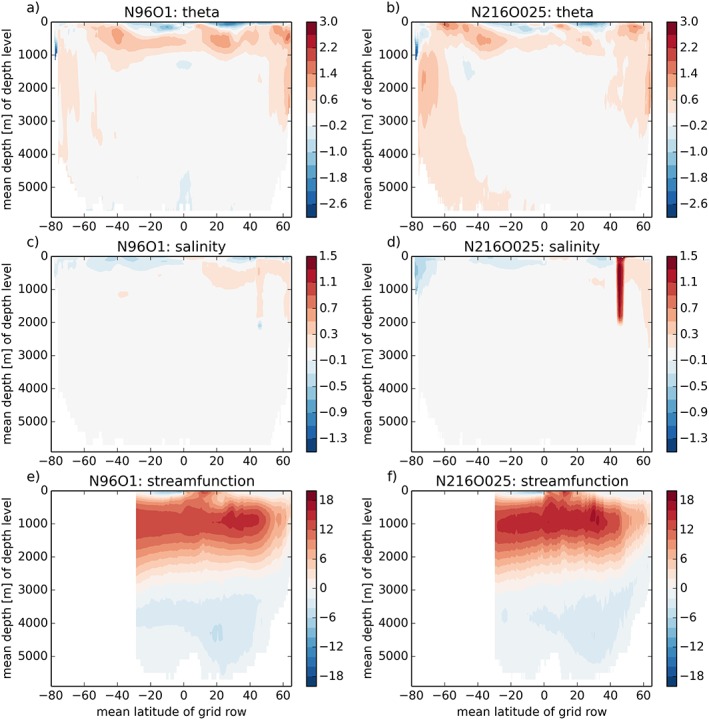
Zonally averaged time‐mean bias (against EN4) of potential temperature (a, b; K) and salinity (c, d) for N96ORCA1 (left column) and N216ORCA025 (right column). Panels (e) and (f) show the overturning stream function (Sv) in the Atlantic basin.

The zonally averaged temperature bias (Figures 7a and 7b) of the two models shows their different behavior with regards to ocean heat uptake. A significant ocean heat uptake is expected in PD simulations because of the positive incoming net radiation at TOA. In N96ORCA1 (Figure 7a), this heat is mostly found above 1,000 m depth, transported northward and eventually downward by the Atlantic meridional overturning circulation (AMOC). In N216ORCA025 (Figure 7b), the warm bias in the upper 1,000 m is smaller, but there is a warm bias throughout the entire depth of the Southern Ocean. We suggest that this Southern Ocean deep warm bias is a result of the warm bias at the surface.

The zonally averaged biases are smaller for salinity. In N96ORCA1, there is a saline bias in the warm subsurface layer on the NH (Figure 7c). N216ORCA025 is comparatively fresh in the upper layers of the high‐latitude Southern Ocean (Figure 7d); the relation to the Antarctic coastal current present at this resolution will be discussed in a companion paper. At both resolutions, there is a positive salinity bias in the upper 2,000 m around 44°N. This stems from a high salinity in the Black Sea whose circulation with the Mediterranean through the Dardanelles is very confined in the model geometry and more so in N216ORCA025 with its higher resolution—hence the larger Black Sea salinity bias here.

**Figure 8:**
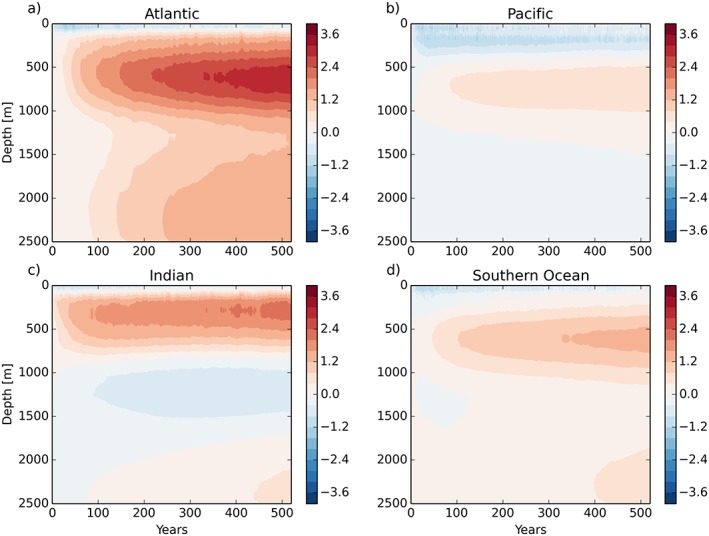
Ocean temperature bias (against EN4) per basin as a function of depth and time in N96ORCA1. Temperature scale is in kelvins. All three basins (a, b, c) are separated from the (d) Southern Ocean at 33°S.

Turning to the depth‐time structure of the temperature bias in N96ORCA1 (against the EN4 climatology), in Figure 8 we find that in all basins there is a subsurface warming between about 200 m and 1,000 m depth (while extending less far up in the Pacific). This warming trend is strongest in the Atlantic and Indian Oceans, and after a few centuries the deep Atlantic warms too (the warm bias being transported downward and then southward by the AMOC). In the Pacific and Southern Oceans the warming has a smaller vertical extent and a much slower rate; in both basins the top few hundred meters of ocean water is colder than in observations, with only weak trends. For the Southern Ocean, this holds in spite of the warm bias at the surface in large parts. The combination of a too cold mixed layer and too warm subsurface waters indicates a too small global vertical temperature gradient, a common feature in CMIP5 models (Kuhlbrodt & Gregory, 2012).

**Figure 9:**
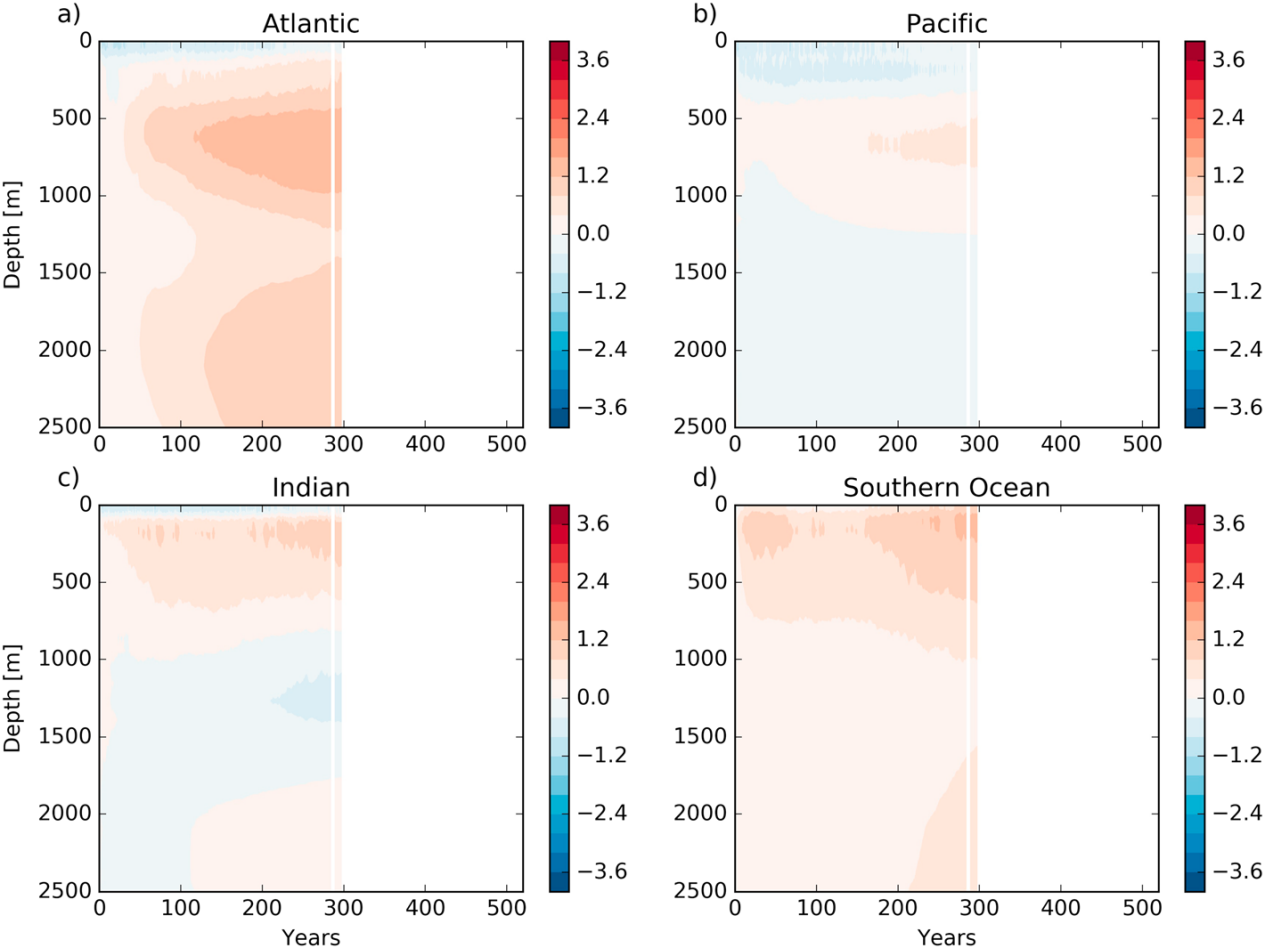
Ocean temperature bias (against EN4) per basin as a function of depth and time in N216ORCA025. Otherwise as Figure 8. The white vertical line masks one year where the output of the simulation was corrupted (but not the simulation itself).

The time‐depth signal of the warm bias in N216ORCA025 (Figure 9) develops in a slightly different way. As discussed above, there is more warming in the Southern Ocean, especially in the uppermost 500 m, while the warming rate in the other basins is less than in N96ORCA1. Note that the initial cooling in the top few hundred meters in the Pacific is smaller too.

**Figure 10:**
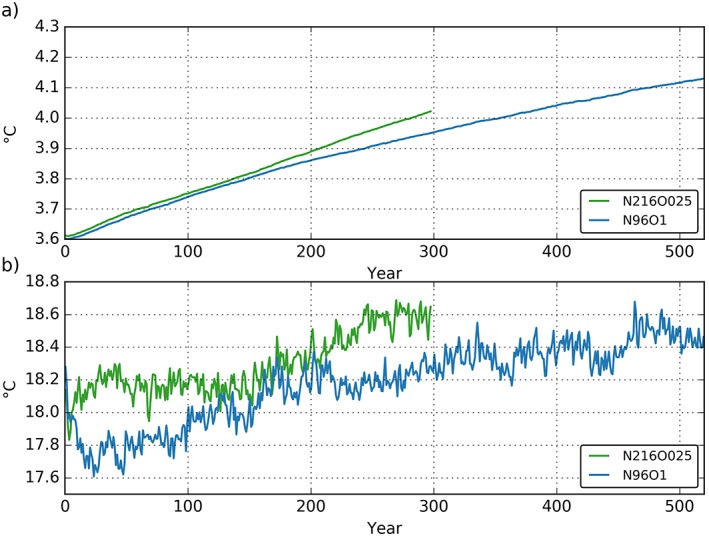
Annual mean time series of (a) global volume‐averaged temperature and (b) sea surface temperature. For years 20 to 100, the rate of change of global volume‐averaged temperature is 0.14 K/century for both N96ORCA1 (blue) and N216ORCA025 (green). The one year in the N216ORCA025 simulation with corrupted output (cf. Figure 9) has been interpolated for this and the following plots.

The global volume‐averaged ocean temperature (Figure 10a) reflects the net heat uptake of the climate system at the top of the atmosphere (TOA; Figure 1a) which, after the first 150 years, is larger in N216ORCA025 than in N96ORCA1. We suspect that the different mechanisms of heat uptake lead to this discrepancy.

The globally averaged sea surface temperature (Figure 10b) at the two resolutions displays the different spin‐up behavior. An initial cooling in N216ORCA025 is small and lasts for only about 30 years. By contrast, the initial cooling is stronger and much more sustained in N96ORCA1. Here the SST time series passes the PD observed value of 18.3 °C after about 200 years. The initial SST cooling (and AMOC weakening; see below) when spinning up a coupled model from an observational climatology is fairly typical for this resolution, compare HadGEM1 in Banks et al. (2007) and the GFDL ESM2 models in Dunne et al. (2012). For the global mean SST, even the recovery time scale of about 200 years is similar across these models.

The AMOC has a substantial influence on the global climate system because of its interhemispheric northward heat transport (Buckley & Marshall, 2016; Kuhlbrodt et al., 2007). From the RAPID array at 26°N (McCarthy et al., 2015) the volume transport of the AMOC is estimated to be 17.2 ± 0.9 Sv for annual means, where 1 Sv = 10^6^ m^3^/s. The stream functions of the AMOC (Figures 7e and 7f) show that, for the average of the years 50–100, the AMOC is somewhat weaker than observations in N96ORCA1 (maximum 15.2 Sv), while in N216ORCA025 it is very close to observations (maximum 16.9 Sv). In line with the stronger deepwater formation in the Nordic Seas discussed above, the overturning cell extends further north in N96ORCA1. Note that the deep overturning cell of Antarctic bottom water is stronger in N96ORCA1 (>2 Sv); this could be related to the Antarctic bottom water being colder in N96ORCA1.

**Figure 11:**
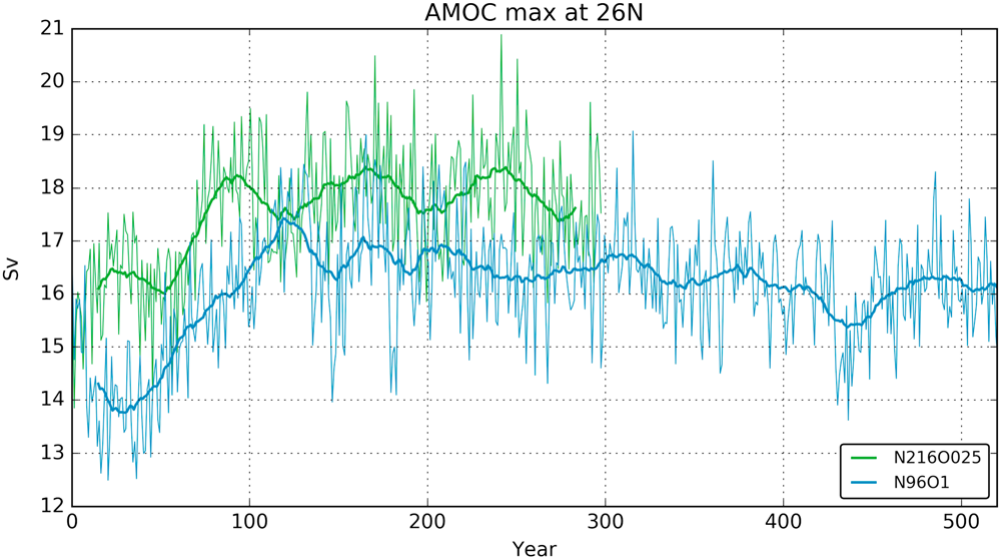
Maximum northward volume transport of the Atlantic meridional overturning circulation (AMOC) at 26°N for N96ORCA1 (blue) and N216ORCA025 (green). Thin lines show annual means, and thick lines a 30‐year running mean.

The time series of the AMOC overturning at 26°N (Figure 11) shows that initially, especially in the first 50 years or so, the AMOC strength in N96ORCA1 is several sverdrups too low while being close to the observed value in N216ORCA025. After the first century, the AMOC is comparatively stable and close to observations at both resolutions. It is worth noting that, when comparing the AMOC as computed using the full velocity field in models to the RAPID estimate, the RAPID method may actually underestimate the full transport by ~1.5 Sv (Sinha et al., 2018).

**Figure 12:**
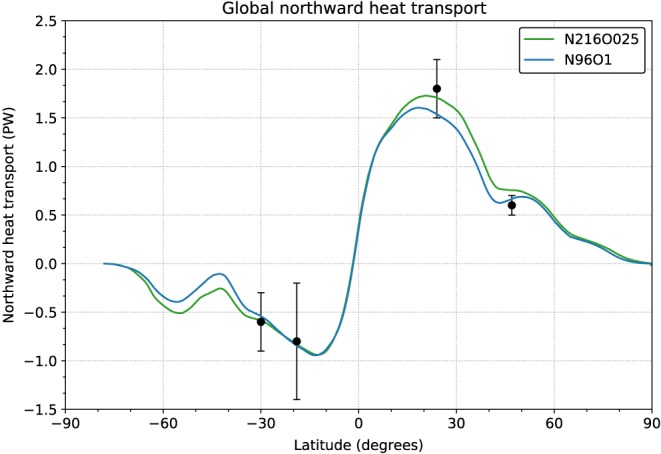
Global northward heat transport. Latitude values are indicative north of 23 N because of ORCA1's two grid poles on the Northern Hemisphere. The dot‐and‐whisker symbols indicate values inferred from observations (Ganachaud & Wunsch, 2003).

In line with the somewhat weaker AMOC in N96ORCA1, the poleward oceanic heat transports are also somewhat smaller compared to N216ORCA025. Figure 12 shows that in three out of four latitudes where observation‐based estimates (Ganachaud & Wunsch, 2003) are available, the higher‐resolution version of GC3.1 matches these more closely. However, at 47°N, N96ORCA1 appears to be closer to observations, while it is within uncertainty estimates at the other locations.

For N96ORCA1, the global northward heat transport has been calculated including the contribution from the parameterized eddy‐induced transport. Nevertheless, from Figure 12 it is clear that at this resolution the poleward heat transport on both hemispheres is smaller than in the eddy‐permitting N216ORCA025 resolution. In future work we will assess the reason for that.

**Figure 13:**
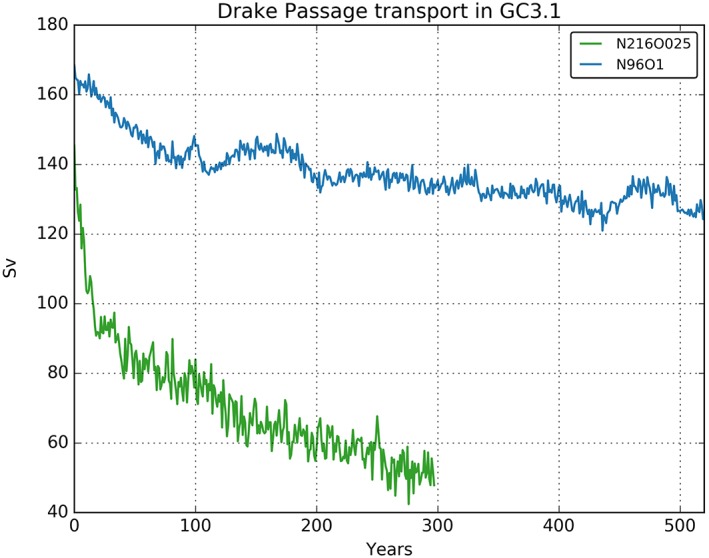
Drake Passage transport in the present‐day simulations of N96ORCA1 (blue) and N216ORCA025 (green).

The ACC is in balance with the meridional density gradient in the Southern Ocean and is partly driven by the wind stress. We show the ACC, diagnosed as the volume transport through Drake Passage, in Figure 13. For comparison, the latest observational estimate is 173 ± 11 Sv (Donohue et al., 2016), while previous estimates could not fully and consistently assess the barotropic component of the Drake Passage transport and hence were markedly smaller, for instance, 137 ± 8 Sv in Cunningham et al. (2003). By these measures, the ACC in N96ORCA1 is within observational estimates in the first decade or two into the PD simulation, while a small but persistent downward trend lets the ACC appear as too weak afterward. Note, however, that in past climate model intercomparisons (Kuhlbrodt et al., 2012; Meijers, 2014) a very wide intermodel spread was found (155 ± 51 Sv for CMIP5). Seen in this light, the ACC strength in N96ORCA1 remains reasonable. In N216ORCA025, by contrast, the ACC quickly slows down to less than 100 Sv and then decreases further to about 50 Sv. This is a consequence of heat uptake poleward of 60°S throughout the entire water column (Figure 7), not being density compensated by a trend in salinity.

### 3.3 Sea Ice

**Figure 14:**
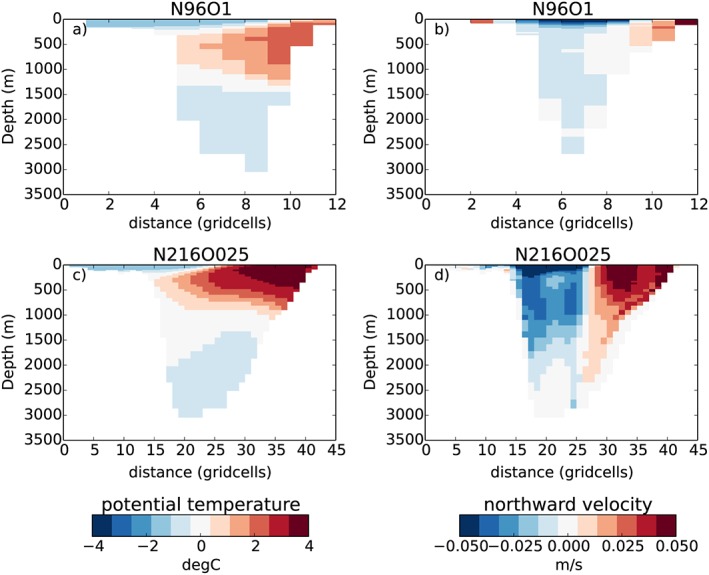
Cross sections of the Fram Strait annual mean ocean temperature (a, c) and velocity (b, d) for N96 ORCA1 and N216 ORCA025.

Sea ice thickness and concentration directly respond to local forcing, including atmospheric and oceanic heat transport. The heat transport of Atlantic water through the Fram Strait determines the Arctic Ocean temperature in the top 300 m. Warm water at this depth can influence the sea ice through the ocean‐to‐ice heat flux which in turn can moderate the basal growth in winter (Keen et al., 2013). The Fram Strait heat transport in HadGEM3 GC3.1 varies with ocean model spatial resolution, since (a) Fram Strait is only 12 grid points wide in NEMO ORCA1 (Figure 14a); and (b) NEMO ORCA1 uses a parameterization for mesoscale eddies (see section 2), as opposed to NEMO ORCA025. This parameterization tends to smooth out ocean fronts and so reduces the flow through straits (Figure 14). The Fram Strait heat transport in ORCA025 varies interannually in the range 26–49 TW, in good agreement with the observationally derived value of 28–46 TW (Schauer et al., 2004). However, the equivalent ORCA1 heat annual mean transport is 3–5 TW, which, being a factor 10 smaller, results in colder Arctic waters year‐round. As a consequence the winter mixed layer entrainment of deeper warm water will not result in warmer water in contact with the ice, and in spring the onset of enhanced basal melt will be absent, both effects causing annual mean ice to be thicker than observed.

To compensate for the lack of basal melt, the surface albedo of snow covered ice is reduced from 0.98 to 0.96 in the visible spectral band and from 0.70 to 0.68 in the near infrared spectral band. The albedo reduction of 0.02 balances the desired effect in the Arctic Ocean with a tendency toward increased ocean heat uptake. Such an albedo reduction allows a slightly earlier onset of snow melt and subsequently longer sea ice melt season. A greater summer melt can thus offset the reduced basal sea ice melt to match the observed Arctic sea ice thickness. The values of snow albedo, in both model resolutions, lie within the observational uncertainty.

**Figure 15:**
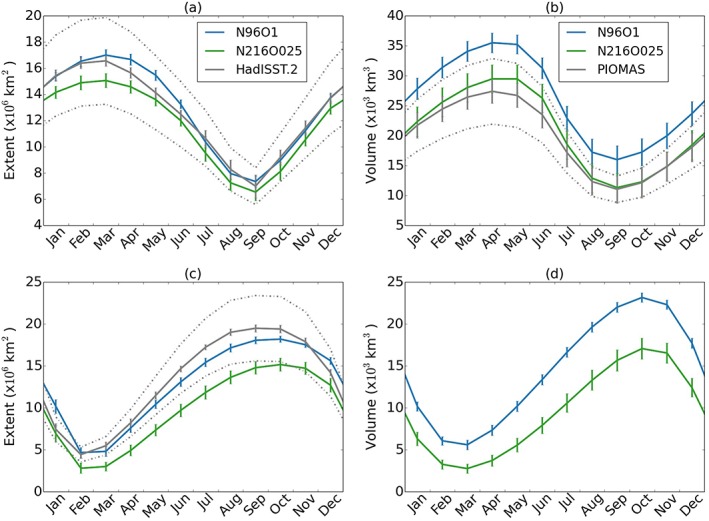
Sea ice mean annual cycles for N96ORCA1 (blue) and N216ORCA025 (green). (a) Arctic ice extent, (b) Arctic ice volume, (c) Antarctic ice extent, and (d) Antarctic ice volume. In (a) to (c), the model data are assessed against observations (solid gray, with dashed lines for a ±20% envelope around the mean following Wang and Overland, 2012). The bars indicate ±1 standard deviation from variability on interannual and longer time scales. The observations are HadISST.2 for sea ice extent and PIOMAS (Schweiger et al., 2011) for Northern Hemisphere ice volume.

Both the high‐ and low‐resolution models are in good agreement with the seasonal Arctic sea ice extent (Figure 15) when compared with the HadISST.2 sea ice analysis (Titchner & Rayner, 2014). In the Southern Ocean, N96ORCA1 shows a better agreement because the Southern Ocean warm bias (Williams et al., 2017) is less severe than at high resolution (cf. section 3.2). The sea ice volume in N96ORCA1 is larger than in N216ORCA025 (Figure 15). In the Antarctic this is solely associated with the greater ice extent, whereas in the Arctic the thicker ice means that more ice survives the summer melt season becoming thicker multiyear ice.

### 3.4 Atmosphere

**Figure 16:**
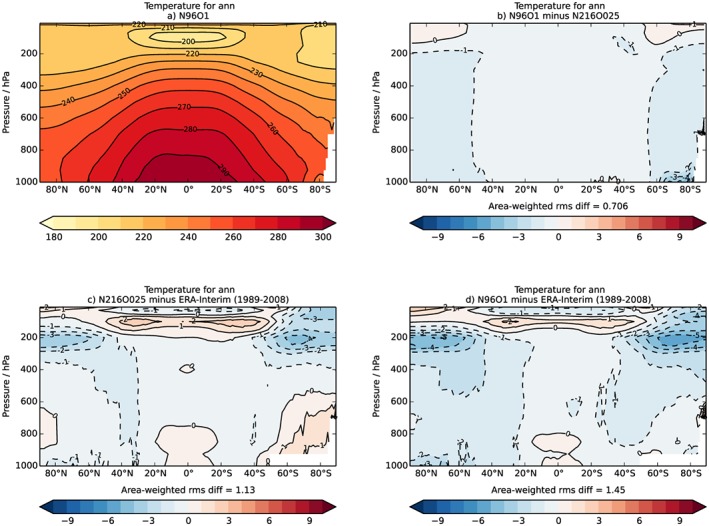
(a and b) Zonally averaged annual mean air temperature in Kelvin. (c and d) The bias against ERA‐Interim (Dee et al., 2011).

For the evaluation of the atmospheric component of N96ORCA1, we compare 50‐year means (years 50 to 100) against the equivalent from N216ORCA025, as in the previous sections. In the troposphere, N96ORCA1 is generally somewhat colder than N216ORCA025 (Figure 16). Akin to the SST (Figure 4), this leads to a cold bias in the NH, while the warm bias in the SH is much smaller in N96ORCA1. At tropopause levels, it is again N96ORCA1 that is colder, by ~1 K.

**Figure 17:**
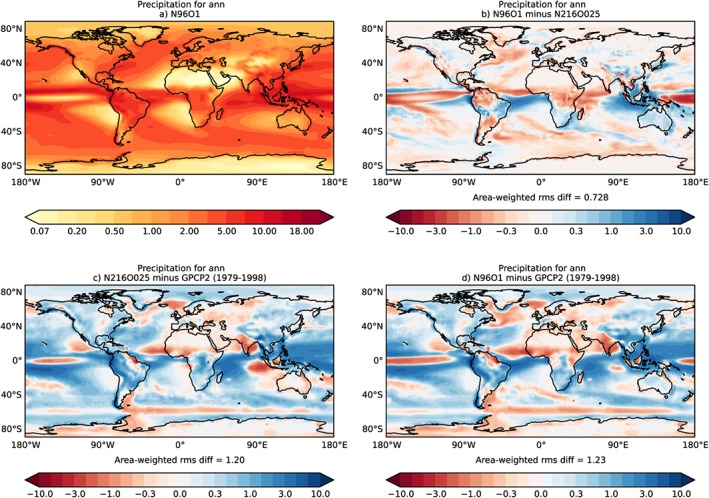
Annual mean precipitation climatology. All values in millimeters per day. (a) Absolute values in N96ORCA1; (b) difference between N96ORCA1 and N216ORCA025; (c) error in N216ORCA025 against observations (GPCP2; Adler et al., 2003); and (d) error in N96ORCA1 against observations.

For global mean precipitation, the largest biases are in the region of the Intertropical Convergence Zone. The geographical pattern of the biases is mostly resolution independent (Figure 17). Because of a misrepresentation of the position of the Intertropical Convergence Zone and its seasonal cycle, some tropical regions are too dry (equatorial Pacific, northern tropical Atlantic and Africa, and the Indian subcontinent) while others are too wet (western tropical Pacific and western tropical South America). Some of the equatorial dry biases are stronger in N96ORCA1 (Figure 17b). The annual global mean precipitation in N96ORCA1 is 3.07 mm/day (3.13 mm/day in N216ORCA025 or 2% more). These values are somewhat too large compared with observational estimates that tend to be just below 3.0 mm/day (Behrangi et al., 2014; Wild et al., 2012). This excess global mean precipitation is a long‐standing issue with the UM and other global atmospheric models (Collins et al., 2010). Indeed, several of the regional annual mean precipitation biases found in N96ORCA1 are common to CMIP5 climate models in the ensemble average (Flato et al., 2013), for instance, the biases over the oceans mentioned above, the dry bias over the Indian subcontinent, or the dry‐wet dipole over Siberia.

**Figure 18:**
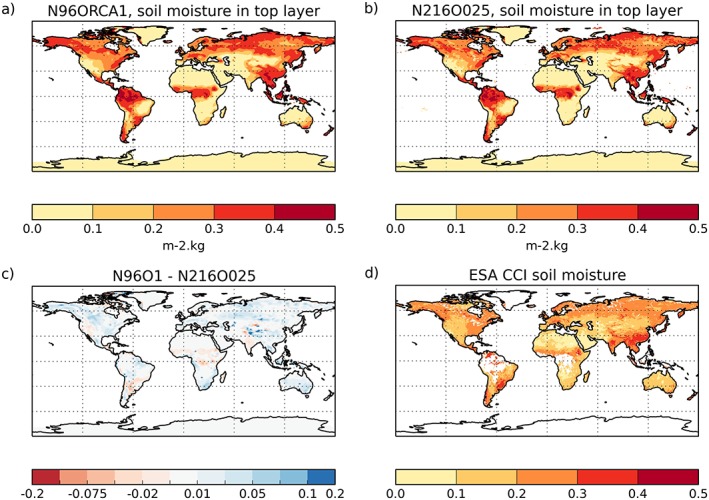
Volumetric soil moisture (m^3^/m^3^) in the top soil layer of N96ORCA1 (a), N216ORCA025 (b; both 50‐year means), and in satellite observations (d; 20‐year mean). The difference between the two models is shown in (c). Shown are boreal summer means (June‐July‐August).

As an indicator for the representation of land surface processes in N96ORCA1, we use the volumetric soil moisture. Figure 18 shows a comparison for the NH summer between the 50‐year mean from the two resolutions and the remotely sensed soil moisture (1996–2015 average) from the combined ESA‐CCI product (Dorigo et al., 2017; Gruber et al., 2017; Liu et al., 2012). Both resolutions capture the global patterns reasonably well (for instance, dry Sahara and wet NH midlatitudes) while they span a wider range of values than the observations: the dry regions are drier and the wet regions wetter than in the ESA‐CCI data. The N96ORCA1 simulation appears to have slightly wetter soil (Figure 18c), mostly where the simulations show a too high soil moisture to begin with. We suspect that differences in precipitation (cf. Figure 17) rather than systematic differences in the land‐surface model are responsible. While most of the wettest regions in the tropics are masked out in the ESA‐CCI data (because of the high cloud cover), the overestimation of high soil moisture is manifest in Southeast Asia and in midlatitude South America. It is likely that this is a result of the excess precipitation in these regions (Figure 17).

The gross primary productivity of the land‐based ecosystems, in the global total (carbon), is 119 ± 6 Pg/year in a recent observation‐based estimate (Jung et al., 2011). As simulated by JULES in N96ORCA1, it is 111.1 Pg/year, slightly below the observed value. This might be related to the low soil moisture in some subtropical regions discussed above. In the N216ORCA025 simulation, it is even lower (102.0 Pg/yr), possibly related to the lower soil moisture.

### 3.5 Coupled Modes

**Figure 19:**
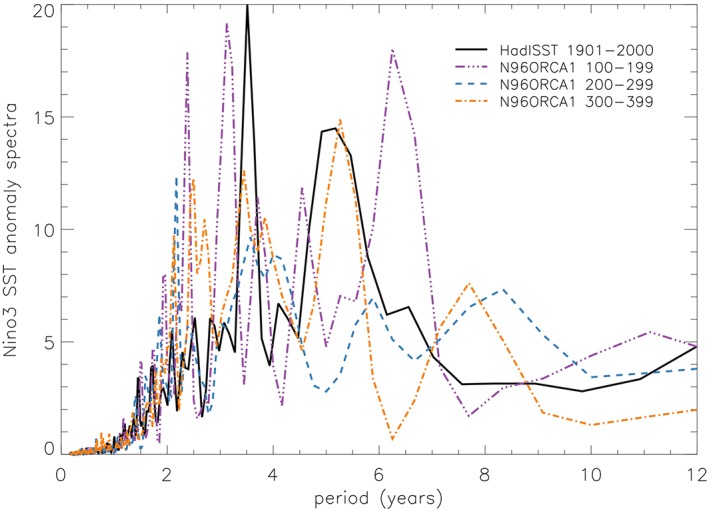
Niño3 (90°–150°W, 5°N–5°S) SST anomaly power spectra for three consecutive 100‐year time slices of N96ORCA1 (purple, dash/triple dot; blue, dashed; and amber, dash‐dotted) and 100 years of observations (HadISST; black, solid). SST = sea surface temperature.

El Niño/Southern Oscillation (ENSO) is the largest natural mode of interannual climate variability in the tropics; oscillations between warm and cold phases occur on a 3‐ to 7‐year time scale, and the effects are felt worldwide. A realistic simulation of ENSO is an important goal for climate models. Power spectra for the central east Pacific region, Niño3, for three 100‐year time slices of N96ORCA1 PD and observations show that the model has broadly the correct dominant frequencies, although there may be slightly more power in the 2‐ to 3‐year time scale than observed (Figure 19). As has been seen in other models, there are multidecadal variations (e.g., Wittenberg et al., 2006) with ENSO being more active here, on the time scales between 2.5 and 7 years, in the first and third centuries (purple and yellow) than the second (blue).

A selection of ENSO metrics describing model performance is shown in Table 2. These can be compared with Table 3 of Williams et al. (2017) for recent HadGEM3 configurations and Bellenger et al. (2014) for CMIP3 and CMIP5 models. The amplitude of SST variability (M1 and M2) is well reproduced for central and central east equatorial Pacific regions although the phase locking of the signal to be at a maximum in early boreal winter and a minimum in spring (M4) is a little weak compared with observations. The variance of precipitation in the west Pacific (M5), which gives an indication of the impact on the large‐scale circulation, is reasonable. A key mean state difference between N96ORCA1 and N216ORCA025 is the annual mean equatorial cold bias evident in N96ORCA1 (Figure 4), a bias common to many climate models. There is also an enhanced warm bias in the far eastern Pacific in N96ORCA1—note that these two biases cancel for the Niño3 region (M6). In addition, N96ORCA1 also has overly strong easterlies in the west Pacific (M7). Overall, N96ORCA1 compares favorably with CMIP5 models.

**Table 2:** Selection of ENSO Performance Measures

	M1 σSST‐N3	M2 σSST‐N4	M3 time scale of SST‐N3 power	M4 (σSST‐NDJ)/(σSST‐MAM)	M5 σPPTN‐N4	M6 SST‐N3	M7 TAUX‐N4
N96ORCA1	0.83	0.58	2–7	1.3	2.1	25.8	−0.040
Observations	0.79	0.54	**3.5**, 5.2	1.6	2.3 (2.7)	25.7	−0.034

*Note*. M1 and M2 are the standard deviation of monthly SST anomaly for regions Niño3 (90–150°W, 5°N–5°S) and Niño4 (160°E–150°W, 5°N–5°S; K). M3 shows power spectrum time scales for monthly Nino3 SST anomaly (years, dominant time scale is in bold), M4 is a seasonality metric defined as the ratio of November to January and March to May standard deviation of Niño3 SST anomaly, M5 is the standard deviation of precipitation anomaly for Niño4 (mm/day), M6 is annual mean SST for Niño3 (K), and M7 is the annual mean zonal wind stress for Niño4 (N/m^2^). SST observations are HadISST, 1901–2000 (Rayner et al., 2003), precipitation is GPCP v2.2, 1979–2013 (Adler et al., 2003; value in brackets from CMAP, 1979–2015; Xie and Arkin, 1997), and wind stress is Southampton Oceanography Centre (SOC) climatology (Josey et al., 1998). ENSO = El Niño/Southern Oscillation; SST = sea surface temperature.

The Atlantic Multidecadal Variability (AMV) is a natural variability mode in North Atlantic SST with a period of roughly 65 to 80 years (Deser et al., 2010). It has a strong influence on precipitation over land masses around the Atlantic basin and Atlantic hurricane activity. The AMV index is defined as the difference between the area‐average SST anomaly over the North Atlantic (0°–60°N) and the global SST anomaly (Buckley & Marshall, 2016).

**Figure 20:**
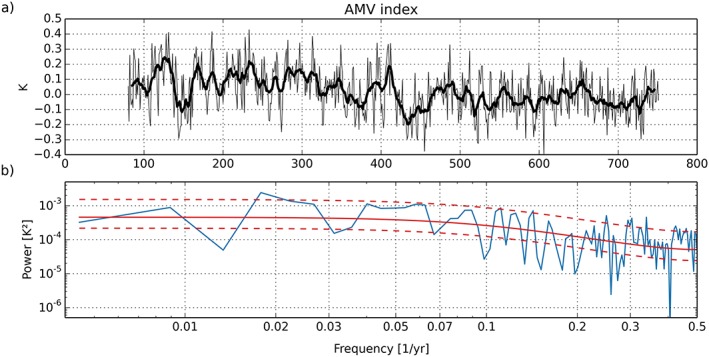
(a) AMV index from years 80 to 752 of the N96ORCA1 present‐day control simulation. Annual means are plotted as a thin line, and the thick line shows an 11‐year running mean. (b) Power spectrum of the AMV index time series, calculated using a fast Fourier transform and a Bartlett window (blue). A theoretical AR(1) spectrum, diagnosed from the autocorrelation coefficient, is plotted in solid red. The dashed red lines show the 95% confidence interval around the AR(1) spectrum. AMV = Atlantic Multidecadal Variability.

Figure 20a shows the AMV index from the N96ORCA1 PD simulation, with the first 80 years removed because of the spin‐up behavior. The power spectrum of the diagnosed AMV time series (Figure 20b) displays significant peaks with respect to a theoretical AR(1) spectrum at frequencies of approximately 0.017 ≈ 1/60 year^−1^ and 0.3 ≈ 1/3 year^−1^. In a power spectrum from the observational data set HadISST (Buckley and Marshall, 2016; Figure 6b), these two peaks are at similar frequencies (1/70 and 1/2.3 year^−1^). This suggests that, in spite of the cold SST bias in the Atlantic discussed above, the N96ORCA1 PD simulation realistically captures large‐scale coupled atmosphere‐ocean Atlantic variability while acknowledging that some of the observed variability may be forced, as opposed to the simulations. This is an improvement compared to a selection of CMIP5 models (Kavvada et al., 2013), which showed a spectral peak around 1/20 year^−1^ that appears not to be present in the observed AMV index.

## 4 Conclusions

HadGEM3 GC3.1 N96ORCA1 is a new, low‐resolution configuration of HadGEM3 GC3.1, the climate model of the UK Met Office in its current release. N96ORCA1 requires 1 order of magnitude less computing power per model year than the medium‐resolution version of HadGEM3 GC3.1, N216ORCA025. At the same time, the PD global climate is represented almost as well as in the medium‐resolution version.

The near‐surface temperature in the simulated PD climate is too low in almost all regions in N96ORCA1. In the North Atlantic and adjacent regions, this exacerbates a cold bias that, to some extent, the medium‐resolution version shows as well. This cold bias might stem from the inability of the 1° ocean model to correctly represent the pathway of the North Atlantic Current; this is a typical deficiency of 1° ocean models.

In the midlatitude to high‐latitude SH it is beneficial that N96ORCA1 has a colder surface (or near‐surface) temperature because this mitigates the Southern Ocean warm bias that both resolutions of HadGEM3 GC3.1 display. In consequence, the representation of Southern Ocean climate in N96ORCA1 has smaller errors than N216ORCA025 in that, for instance, the volume transport of the ACC and the seasonal cycle of sea ice extent are much closer to observations in N96ORCA1.

For the spin‐up of the simulated climate state when starting a simulation from an observational climatology for the ocean, centuries‐long time series of global radiation at the top of the atmosphere suggest that after about 300 or 400 years the modeled climate state in N96ORCA1 reaches an equilibrium, in similarity to other coupled climate models (Banks et al., 2007; Dunne et al., 2012). An initial loss of heat from the ocean's mixed layer, during the first few decades of the model simulation, is followed by a continuous ocean heat uptake—mostly in the top 1,000 m—whose rate decreases over the first two or three centuries in N96ORCA1.

N96ORCA1 has been developed with a high degree of traceability to the medium‐resolution version N216ORCA025, meaning that any changes in the model's parameterizations are directly justified by the coarser horizontal resolution, for example, for horizontal viscosity and isopycnal tracer diffusion in the ocean component. However, there is a single exception. In N96ORCA1, not enough warm Atlantic water is transported northward into the Arctic Ocean. This leads to the water temperature being too low in the subsurface Arctic, and hence to insufficient bottom melt of sea ice, and thus to too thick sea ice in comparison with observations. This is a consequence of Fram Strait being only 12 grid points wide—as above, a deficiency that is hard to correct in a 1° ocean model. In order to obtain a realistic sea ice thickness in the Arctic Ocean, we have slightly reduced the albedo for snow on sea ice. This reduction, by 0.02, is still well within the observational uncertainty of this quantity.

The ocean component of N96ORCA1, *shaconemo* NEMO ORCA1, is being used in a number of other current coupled climate models and ESMs, providing an opportunity to analyze the impact on oceanic circulation (and biogeochemistry) of different atmosphere and sea ice models coupled to the same ocean model.

HadGEM3 GC3.1 N96ORCA1 is the physical core of UKESM1 and is used for many CMIP6 experiments, including CMIP‐DECK. Because of its computational efficiency and good scientific performance, it lends itself to multicentury simulations and ensemble experiments.

## Appendix A

We conducted a number of sensitivity tests with NEMO parameters in N96ORCA1 to address the cold bias in the North Atlantic. This was done separately from the sensitivity tests with CICE parameters, mentioned in section 3.3 above. Below we discuss briefly the impact of parameter variations in NEMO on ocean temperature and circulation.

The isopycnal diffusivity was increased by 25% and 50% in two test simulations (baseline value: 1,000 m^2^/s). In 20‐year means around year 50 of the simulations, a marked increase of SST in large parts of the global ocean was found (around 0.25 K in the global average for the 50% case and around 0.1 K in the 25% case). A penetration of this signal to depths beyond the mixed layer did not occur in this relatively short simulation. The AMOC transport at 26°N was not discernibly affected. Because the warming in these test simulations did not affect the very cold region in the northwest Atlantic, these parameter changes were not adopted.

In NEMO's TKE scheme, several parameters were varied to reduce turbulent exchange between the (too cold) surface waters and the (too warm) subsurface waters; we discuss a selection of these experiments below. As before, 20‐year means around year 50 of the simulations were analyzed. The impact on AMOC transport was either very small or not discernible.

A reduction of the vertical eddy viscosity by 10% (NEMO parameter *rn_ediff* = 0.09) lowered the SST in the North Atlantic by about 1 K, with little effect elsewhere.

A reduction of the near‐inertial wave breaking scaling by 20% (*rn_efr* = 0.04) led to a moderate SST warming in some parts of the midlatitude to high‐latitude NH, with little effect elsewhere.

Changing the latitudinal dependence of the length scale for the exponential decrease of TKE penetration by two thirds in the high‐latitude Southern Ocean (*nn_htau* = 0; length scale reduced from 30 m to 10 m) yielded a strong warming signal in SST, especially in the SH (>1 K) where the warm bias is worsened, while the reduction of MLD reduces the bias in this quantity. However, there is hardly any warming in northwest Atlantic SST.

Scaling the same latitudinal dependence by a factor of 2/3 everywhere outside the tropics, where this length scale is 0.5 m, means a reduction of the high‐latitude maxima to 20 m in the Southern Ocean and 6.67 m in the NH (*nn_htau* = 4 as used in the present configuration; *zmax_scale* = 2/3). This leads to an SST increase on the global average by ~0.15 K, located mostly in midlatitude regions and the Nordic Seas.

The results of these sensitivity tests are in line with the ocean‐only tests conducted by Calvert and Siddorn (2013). Unlike the isopycnal diffusivity that needs to vary with horizontal grid resolution in any case (Table 1), varying these parameters would have constituted a significant breach of traceability with N216ORCA025—both configurations have the same 75 depth levels—and hence was not pursued.

Viscosity settings in NEMO can have a marked impact on the circulation. Comparing the current version of N96ORCA1, using a Laplacian viscosity of *rn_ahm_lap_0* = 20,000 m^2^/s and free‐slip boundary conditions, with a development version that used *rn_ahm_lap_0* = 10,000 m^2^/s and no‐slip boundary conditions, we found a substantial improvement in the representation of deepwater formation in the Labrador and Irminger Seas (diagnosed as the maximum monthly mean MLD).

We explored the impact of deepening and widening the bathymetry in the region of the overflows in the northeast Atlantic using an earlier version of the N96ORCA1 configuration (UM8.6, NEMO3.4). Only quite significant changes to the bathymetry in Denmark Strait and on the Iceland‐Scotland ridge led to an enhanced AMOC transport at 26°N and higher SST (by 2 or 3 K) throughout the North Atlantic. These effects lasted for about 20 years, after which the differences to the baseline configuration became very small.
